# A green synthesis of zinc oxide nanoparticles using Musa Paradisiaca and Rooibos extracts

**DOI:** 10.1016/j.mex.2022.101892

**Published:** 2022-10-27

**Authors:** GV Lyimo, RF Ajayi, E Maboza, RZ Adam

**Affiliations:** a Restorative Dentistry Department, Faculty of Dentistry, University of the Western Cape, Francie van Zijl Dr, Parow, 7505; b Chemistry Department, Chemical Sciences Building, Faculty of Natural Sciences, University of the Western Cape, Robert Sobukwe Road, Bellville, 7535; C Faculty of Dentistry, University of the Western Cape, Francie van Zijl Dr, Parow, 7505

**Keywords:** Green ZnO nanoparticles, Biosynthesis, Musa Paradisiaca, Aspalathus linearis

## Abstract

This study describes the single pot synthesis of zinc oxide nanoparticles (ZnO NPs) using a mixture of *Aspalathus linearis* and *Musa paradisiaca* for use against the fungi *Candida albicans*. These nanoparticles are known to be one of the most multifunctional inorganic nanoparticles with effective antifungal and antibacterial activity. The synthesized ZnONPs were characterized by a peak at 290 nm in the UV–vis spectrum while HRSEM confirmed rod-shaped nanoparticles. The FTIR data clearly revealed that the extracts contained -OH  functional groups whose role was capping agents during the nanoparticle synthesis. This study also found that the purity of the green synthesised ZnO NPs (GZnO NPs) was 94.4 %, 91.5 %, and 82.1 %, respectively, using XRD, HRTEM, and HRSEM-EDS. The antifungal activity of ZnONPs was tested against *Candida albicans* using the Kirby Bauer method. The maximum inhibition zone observed in the ZnO NPs against *Candida albicans* was confirmed to be 24 mm, a clear indication that the synthesized ZnO NPs have great potential to act as effective antifungal agents.

• Zinc nitrate hexahydrate [Zn(NO_3_)_2_·6H_2_O] was used as the inorganic metal oxide precursor.
• Extracts of banana (*Musa paradisiaca*) peel and tea leaves of Rooibos (*Aspalathus linearis)* infused with Buchu (*Agathosma betulina*) were the organic constituents used as reducing and capping agents during GZnO NPs synthesis.
• Validation of the formed GZnO NPs was done using; Ultraviolet-visible spectroscopy (UV-Vis), X-ray spectroscopy (XRD), Fourier-transform Infrared Spectroscopy (FTIR), High-resolution Transmission Electron Microscopy (HRTEM) and Selected Area Electron Diffraction (SAED), and High-Resolution Scanning Electron Microscopy with Energy-Dispersive Spectroscopy (HRSEM-EDS).

## Graphical abstract

**Figure:**
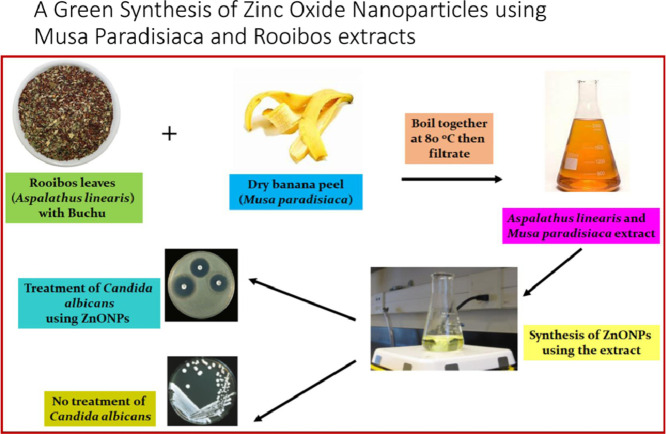

**Specifications table**

**Table:** 

Subject Area	Chemistry
More specific subject area	Green Nanotechnology
Method name	Green synthesis of Zinc Oxide Nanoparticles
Name and reference of original method	[1]. Green synthesis of ZnO nanoparticles by Aspalathus linearis: Structural & optical properties. J. Alloys Compd. 646, 425–430. https://doi.org/10.1016/j.jallcom.2015.05.242
Resource availability	*N.A.*

## Materials and methods

### Armamentarium, reagents, and materials

**Figure 1:**
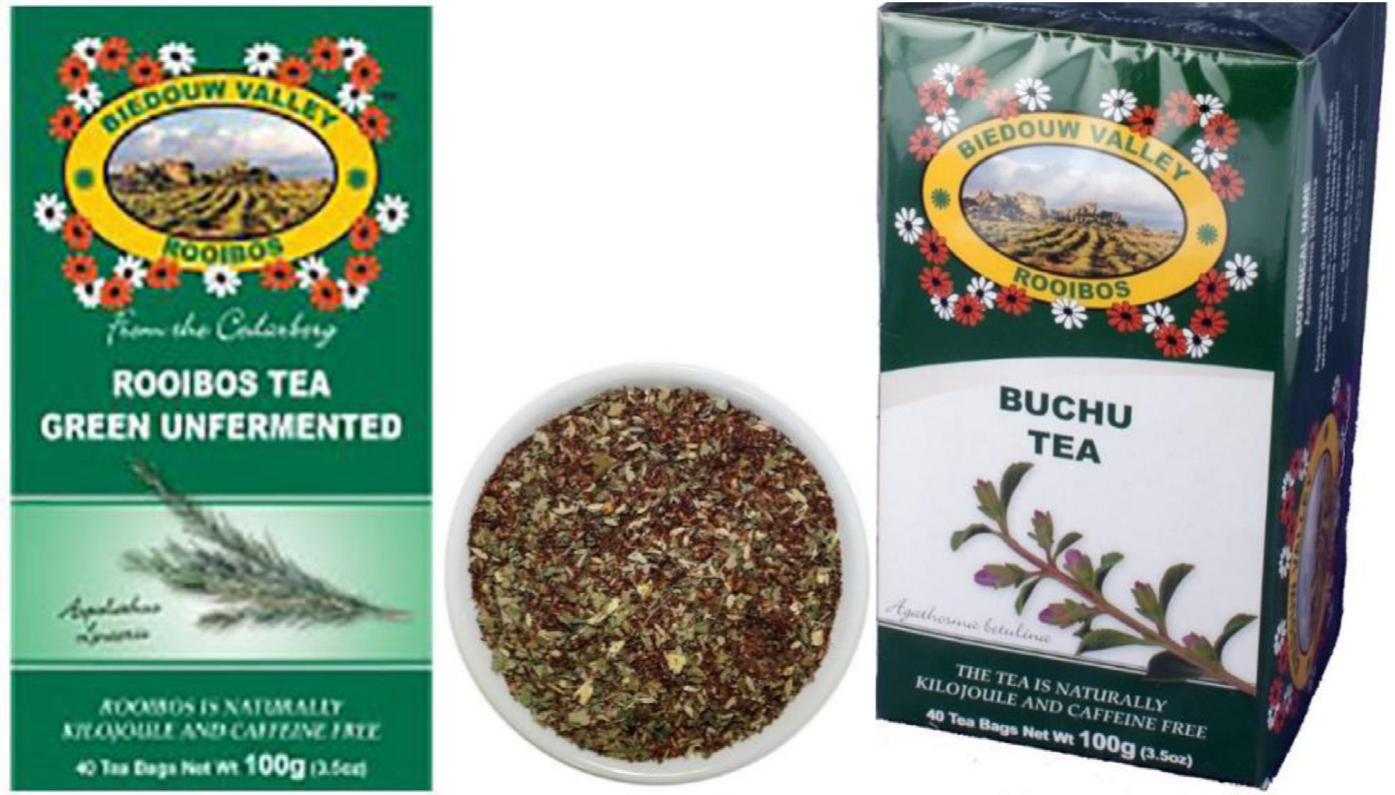
Buchu and Rooibos tea leaves for the preparation of extracts. (https://organiczone.co.za/product/biedouw-valley-rooibos-buchu-tea-40s)

The materials and reagents for the green synthesis of ZnO NPs were distilled water, Rooibos and Buchu tea leaves (commercially available, manufactured by Biedouw Valley Rooibos, Western Cape, SA) (Figure 1 below). Banana peel (collected as remains of organic banana fruit), and zinc nitrate hexahydrate (Zn(NO_3_)_2_·6H_2_O purchased from Merck (Pty) Ltd, South Africa. Essential lab equipment included a magnetic stirrer, a hot plate with a thermometer, glass beakers, aluminium foil sheets, and conical glass flasks.

The Zn was sourced from Zn(NO_3_)_2_·6H_2_O, dissolved in distilled water. The GZnO NPs were manufactured using a mixture of *M. paradisiaca* (BPE), as well as two tea leaf extracts from *A. linearis* (Rooibos) and *A. betulina* (Buchu). Three protocols were adopted and slightly altered to achieve a green single-pot synthesis method. The first two adopted protocols were for the Rooibos and Buchu green tea mixture [1,9] and were combined with a third protocol [6]. Equal volumes (10 ml) of the green teas of Rooibos and Buchu were used as reducing and stabilising agents for the synthesis of green ZnO NPs.

### Preparation of the banana peel extract

According to the protocol by Maruthai et al. [6], the BPE was prepared with slight modifications. The peels were washed and rinsed with distilled water, allowed to dry, and cut into thin slices, after which 100 g of peels were boiled in 300 ml distilled water for 30 min at 70 ᴼC under magnetic stirring. The banana extract was cooled naturally to room temperature (25°C), poured out gently, and centrifuged at 1000 rpm for 15 min. The acquired supernatant (clear extract) was poured out, leaving insoluble fractions of precipitate and macromolecules. The extract was stored (at 4°C) in a well-labelled clean glass bottle as a stock extract solution until further use.

### Preparation of Buchu-infused Rooibos extracts

These extracts were prepared with minor modifications of protocols by Diallo et al., [1] and Thema et al., [9]. In a clean container, 5 g (4:1) of a mixture of Rooibos and Buchu ground tea leaves (Figure 1) was dissolved in 100 ml of distilled water. The solution was then boiled at 80 °C for 2 h under mechanical stirring (1000 rpm), after which it was allowed to cool naturally to room temperature (25°C) and poured out gradually. The solution was then centrifuged at 1000 rpm for 15 minutes, and the supernatant (clear extract) was poured out, leaving insoluble fractions of precipitate and macromolecules. The resultant extract was stored (at 4°C) in a clean storage bottle as a stock extract solution until further use.

### Phytosynthesis of zinc oxide nanoparticles

**Figure 2:**
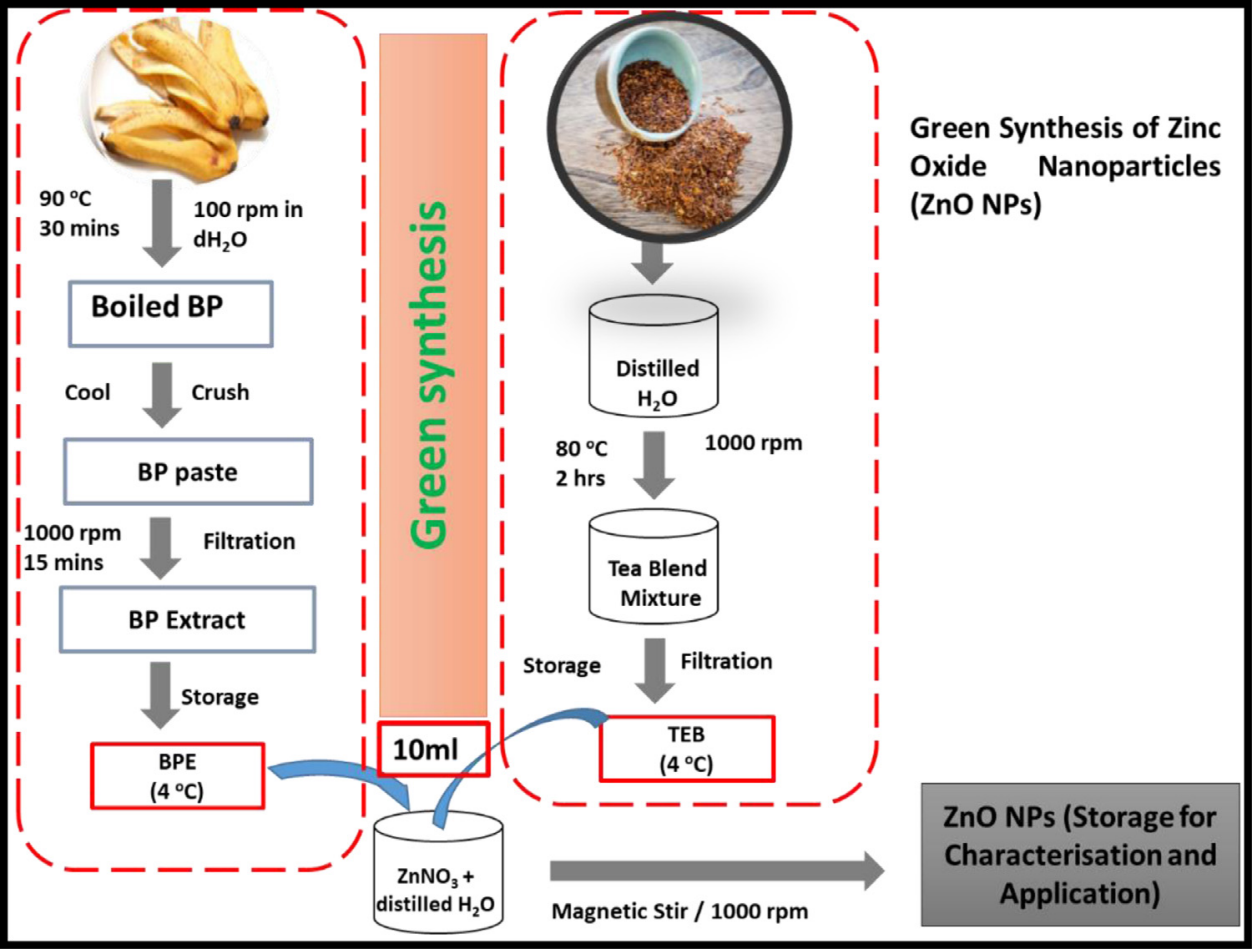
Procedures, materials, and conditions for the phytosynthesis of GZnO NPs.

In order to synthesise the ZnO NPs, a solution of Zn(NO_3_)_2_.6H_2_O (0.1 M) was prepared in 50 ml distilled water in an ultrasonic vibrator to ensure complete dissolution of the salt. Following the completion of this procedure, stock solutions were obtained, and used in the final step of ZnO NPs formulation, as illustrated in Figure 2 below. For this single pot synthesis, the Zn(NO_3_)_2_·6H_2_O solution together with two equal portions (10 ml) of the stock solutions of BPE and Buchu-infused Rooibos extract were added to a sterile conical flask under continuous stirring. The mixture was magnetically stirred at a regular speed while wrapped with an aluminium foil sheet to prevent photoactivation of the zinc nitrate during the synthesis procedure. Temperatures were monitored to remain between 65 and 70°C for 5 h, as illustrated in Figure 2. The resultant mixture was naturally left to gradually cool to 25°C (room temperature). Upon completion of the green synthesis of the ZnO NPs, the colour changes in the mixture were recorded, and the mixture stored in a sterile brown bottle at room temperature for further characterisation and antimicrobial testing.

### Characterisation of nanoparticles

The sample(s) were individually customised before characterisation, to successfully validate the GZnO NPs using the methods listed below in the following forms, either dry the aqueous NPs, dilute the aqueous NPs, formulate pellets, or to use the aqueous NPs *de novo.*

An aqueous sample of GZnO NPs and plant extracts were studied using POLARstar Omega spectrophotometer (BMG labtech, Germany) and the wavelength was set between 200 and 600 nm [5]. A dry sample of synthesized GZnO NPs was obtained by freeze-drying the aqueous solution of NPs using a VirTis Genesis 25 ES Freeze drier (SP Scientific, Warminster, USA). The amorphous sample obtained after freeze-drying was further dried in an oven overnight at 70 °C before assessing the sample under the microscope. The structure and morphology of the GZnO NPs were then interrogated using XRD analysis.

Furthermore, Fourier Transform Infrared Spectroscopy (FT-IR) analysis was also performed according to a previously reported method (using the PerkinElmer spectrum one FT-IR Spectrophotometer, Waltham, MA, USA) [4]. The purified dried nanoparticles were mixed with potassium bromide (KBr) and pressed into a pellet for analysis. Pressed pure KBr was used for background correction. For the High-Resolution Scanning Electron Microscopy (HRSEM) analysis, a dry sample of synthesised GZnO NPs was obtained following freeze-drying of the aqueous solution of NPs. The freeze-dried samples were mounted with double-sided adhesive tape and coated with platinum before analysis. High Resolution Transmission Electron Microscopy (HRTEM) and Selected Area Diffraction Pattern (SAED) were also used to characterize the nanoparticles which were oven dried for 48 hrs to allow better imaging.

#### Antimicrobial activity assay

Kirby-Bauer disc diffusion was used to test the antimicrobial activity of the green synthesized ZnO NPs against *Candidia albicans* (ATCC 90028). The obtained strain was subcultured on BHI agar for a day (24 h) at 37°C and after that stored at 4°C until use. These cultures were refreshed for every subsequent experiment. The reactivation of strains and preparation of the inoculum was performed in a biological class II safety cabinet. Gram staining was performed on the 24 h culture to confirm that the culture sample was Gram-positive oval yeast. For each experiment, the amount of test organism was calibrated as follows: A single colony of the test organism was transferred from 24 hour-old agar plate aseptically into PBS and then homogenised using a vortexer (Eins-Sci E-VM-A Analogue Vortex Mixer, Johannesburg, Gauteng, South Africa). The vortexing was performed to ensure that the yeast cells are randomly suspended in the solution. The resultant homogeneous suspension was then calibrated to a concentration of 0.5 McFarland. The adjusted inoculum was inoculated on Muller-Hinton agar plates. Different volumes of GZnO NPs (50 µl, 100 µL, 150 µl and 200 µl on to sterilized paper discs (6mm in diameter) and placed onto inoculated plates. For comparison, ZnNO_3_ was also tested as well as Chlorhexidine gluconate (CHX) 0.2%. All plates were incubated at 37˚ C for 24 and 48 hrs.The inhibition zone around each disc was measured and was indicative of antibacterial activity for each of the test samples after incubation. This procedure was performed in triplicate.

## Results and Discussion

### GZnO NPs synthesis

**Figure 3:**
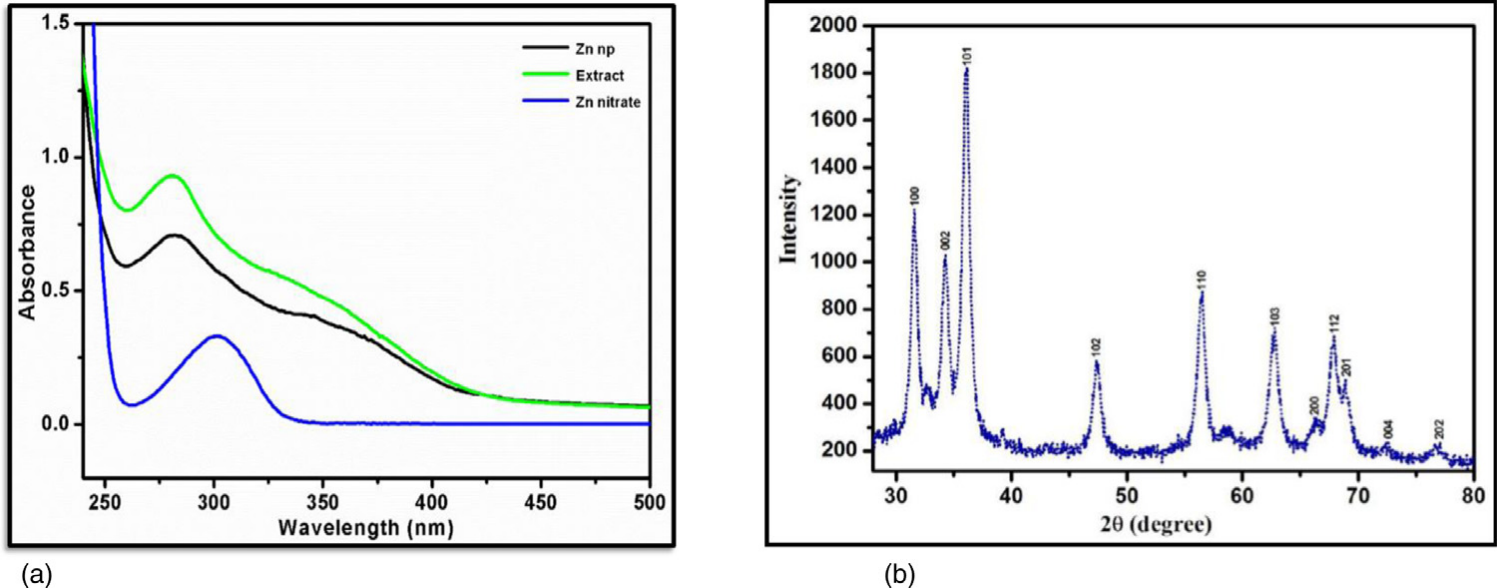
(a) Ultraviolet-visible (UV-Vis) spectrum of the green synthesised zinc oxide nanoparticles (b) X-ray diffraction (XRD) patterns of the green zinc oxide nanoparticles.

In this study, the ZnO NPs formulation was monitored by visual inspection. Observed colour changes were recorded during the entire course of the reaction. Changes are perceived to be attributable to the conversion of the precursor Zn(NO_3_)_2_ to ZnO. The band recorded for a ZnO NPs concentration of 6.538 µg/ 1000 µL from the UV-Vis spectrum was 290 nm (Figure 3), which was similar and within the range of findings reported at bands of 290 to 300 nm for phytosynthesised ZnO NPs [8]. Generally, a broad band in UV-vis spectra is an indication that the size and shape distribution of the nanosized ZnO is somewhat broad, confirming that the sample had a heterogeneous population of ZnO NPsV visible analysis.

**Figure 4:**
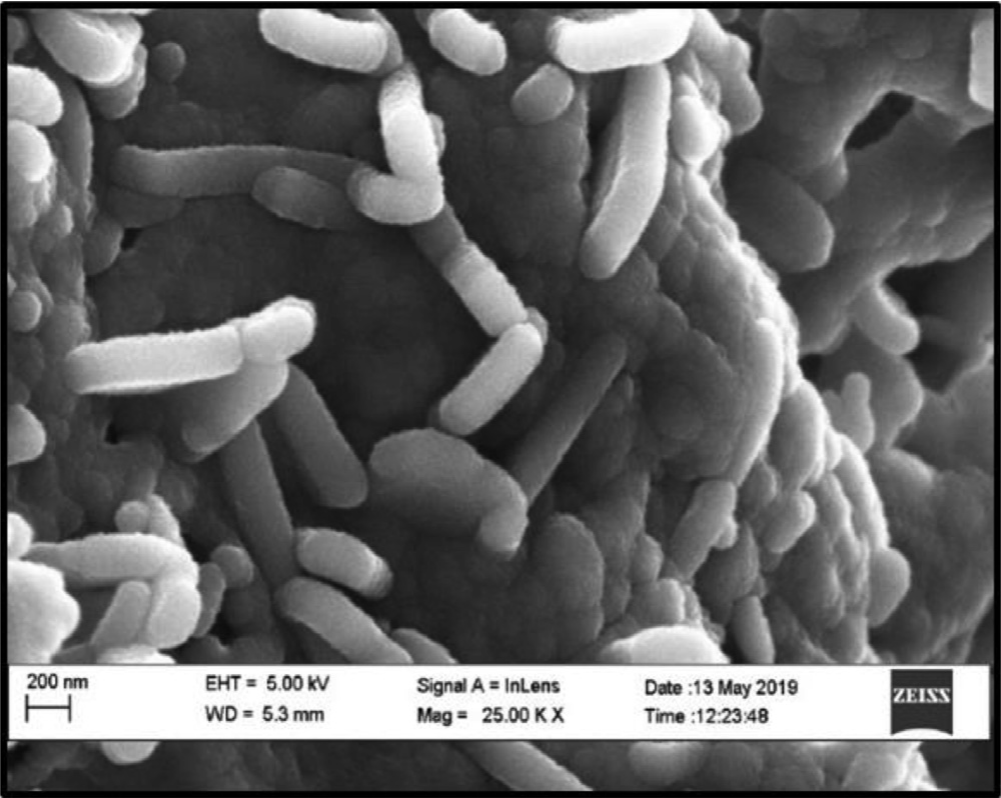
High-resolution scanning electron microscopy (HRSEM) micrographs of the green synthesised zinc oxide nanoparticles at 25.00 K X.

HRSEM interrogation revealed rod-like ZnO NPs for oven dried nanoparticles at 70 °C for 24 Hrs (Figure 4) as apposed to HRTEM which illustrated a crystalline structure for nanoparticles oven dried at 70 °C for 48 Hrs. Several other reports have also described a similar HRSEM presentation of their green synthesis ZnO NPs [2]. The images for HRSEM and HRTEM are different due to imaging challenges experienced in the HRSEM analysis in samples which were oven dried in excess of 24 Hrs and imaging at other magnifications were challenging as well. As such, HRSEM imaging after 24 Hrs at 25.00 K X was the best-recorded image. For the HRTEM imaging, the best image was receieved from nanoparticles which were oven dried for 48 Hrs. The extended drying period caused a drastic change in the morphology of the nanoparticles as illustrated in Figure 6(b) [7]

**Figure 5:**
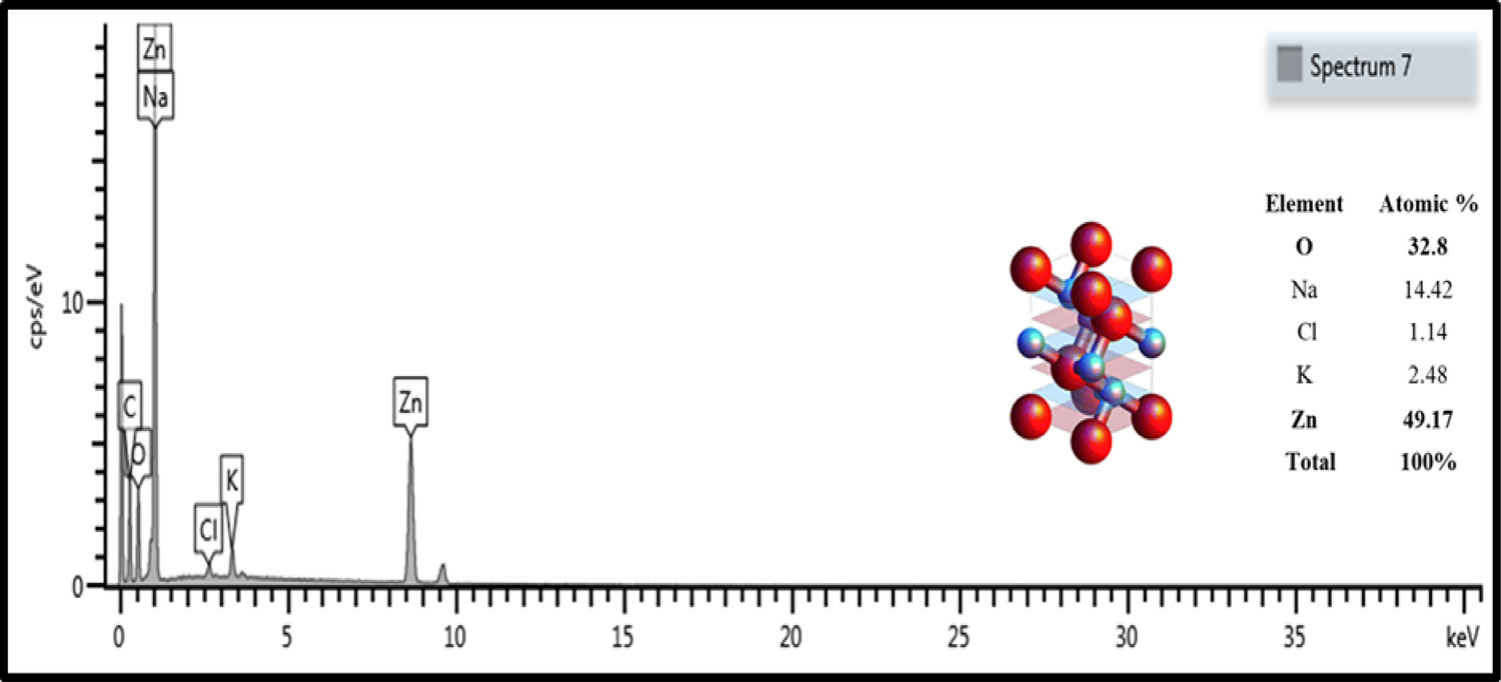
HRSEM spectroscopy and elemental composition, as well as crystalline structure description of the green zinc oxide nanoparticles.

The HRSEM-EDS analysis is a qualitative and quantitative technique employed to study the formulated nanomaterials in this case the green ZnO NPs. It provided the fundamental elemental constituents by the HRSEM-EDS analyses, and the crystalline structure of the green ZnO NPs was further validated (Figure 5).

**Figure 6:**
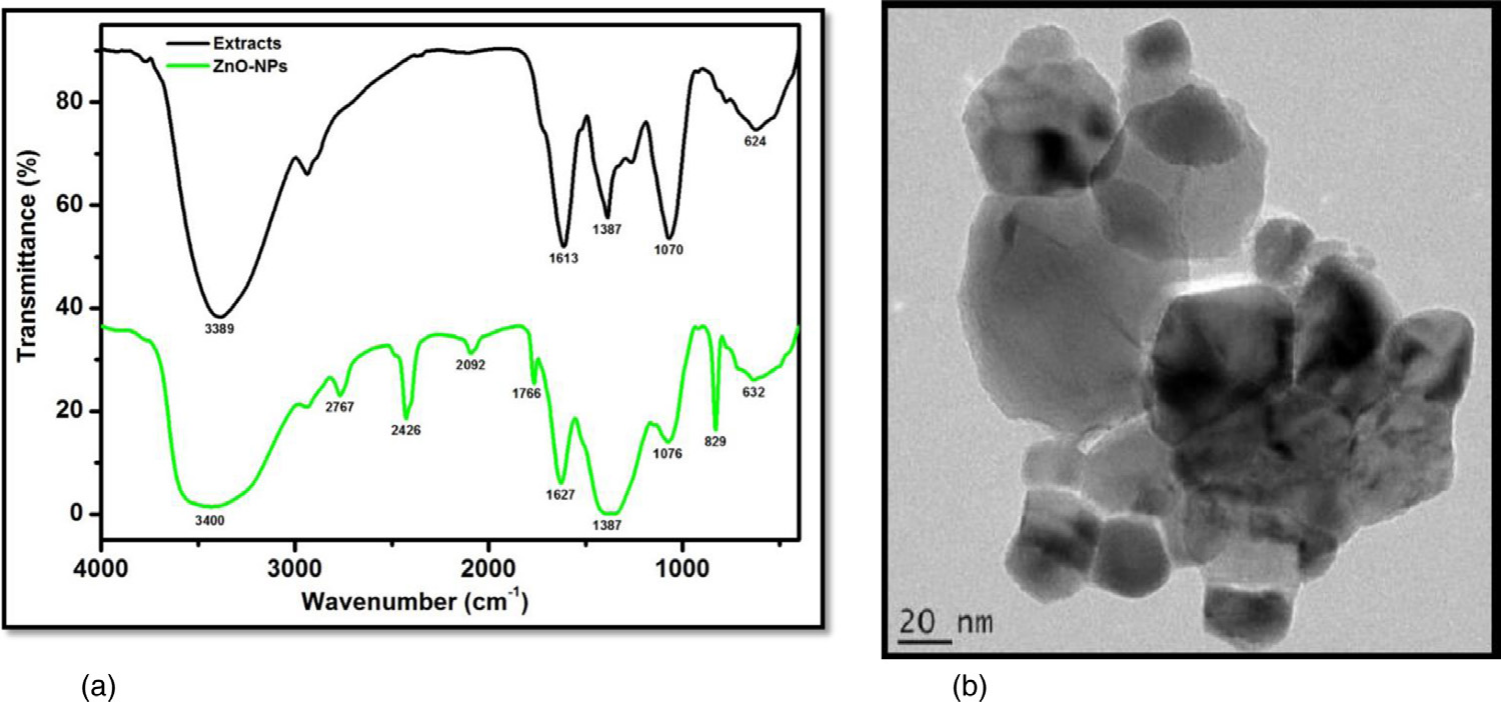
(a) FTIR analysis of the green zinc oxide nanoparticles, in comparison to all extracts combined (b) HRTEM of the green zinc oxide nanoparticles.

FTIR was also performed to re-validate the zincite nature and purity of the synthesized nanoparticles. Figure 6(a), shows the characteristic FTIR band of the pressed ZnO NPs powder in the spectral range of 400 - 4000 cm^−1^. Using the HRTEM image [Figure 6(b)], and HRSEM-EDS, this study found that the purity of the green synthesised ZnO NPs was; 94.4 %, 91.5 %, and 82.1 %, respectively.

### Antimicrobial properties of GZnO NPs

**Figure 7:**
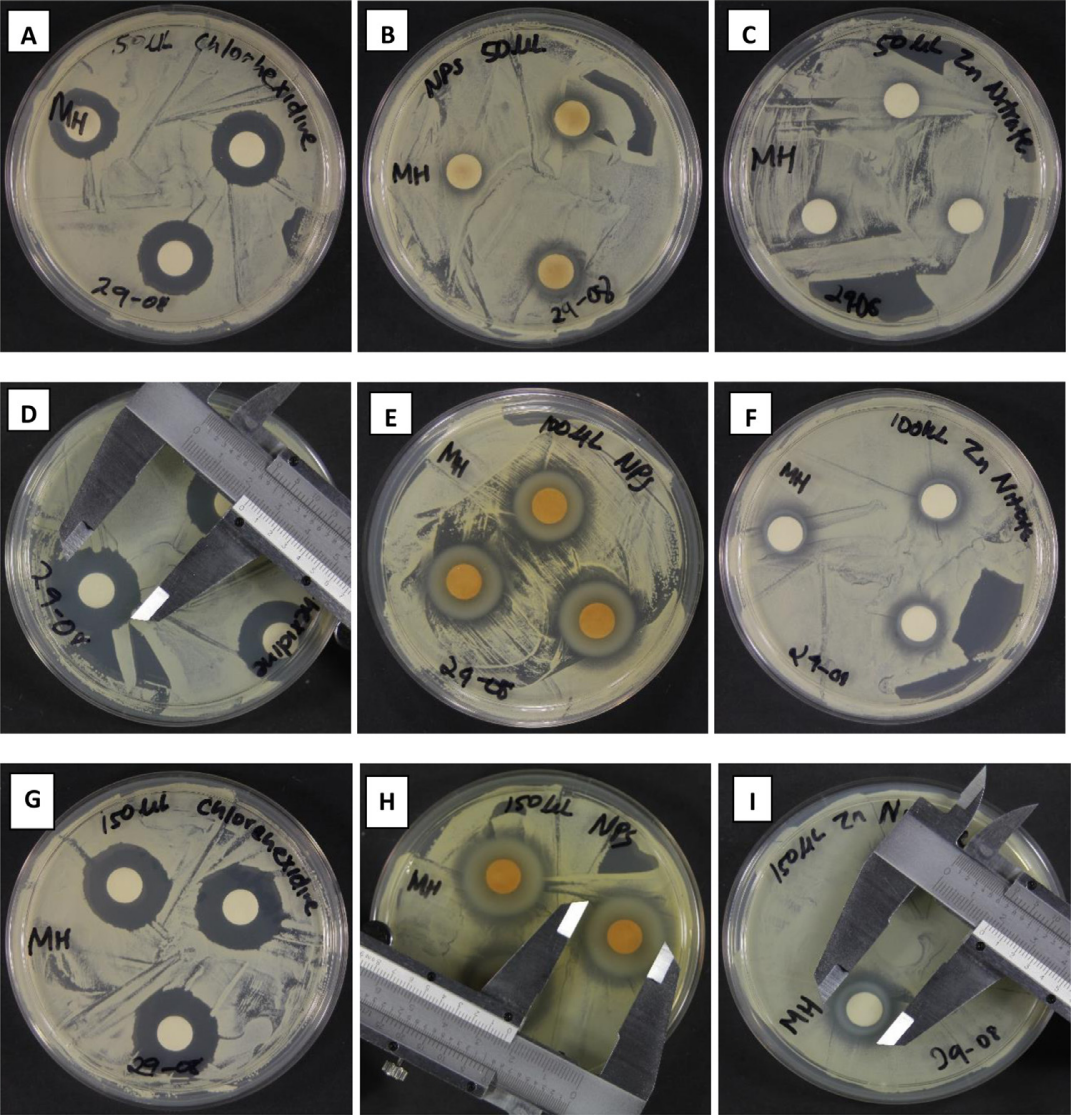
Zones of inhibition for CHX (A, D, G); ZnO NPs (B, E, H); and [Zn (NO_3_)_2_·6H_2_O] (C, F, I).

The diameters of the inhibition zones around the antimicrobial discs, as reflected in Figure 7(b, e, and h), show that these nanoparticles have antifungal activity against C. Albicans. These zones of inhibition increased with increasing volumes (50µl;100µl and 150µl) pipetted onto the sterile discs. Wahab et al., [10], reported similar results with their working concentration of ZnO NPs. This could be due to the increase in NPs concentration with increased volume. The highest differences in the effect between ZnO NPs and CHX were observed at 200 μl. Overall, for all three intervention groups (0.2 % CHX gluconate, green ZnO NPs, and Zn (NO_3_)^2^·6H_2_O), similar trends were observed.

ZnO NPs have distinctive physicochemical properties which are responsible for their antimicrobial properties. These include (a) Zn^2+^ ion release, (b) adsorption, (c) ROS generation, and the intracellular responses in microorganisms of (d) energy metabolism inhibition; (e) lipid peroxidation and cell membrane damage; and (f) DNA replication disruption, and DNA break [3]. Unlike other metal and metal oxide nanoparticles, ZnO NPs have multiple functional mechanisms relating to antimicrobials. ZnO NPs are also able to combat multi-drug resistance based on targeting antibiotic-resistant pathways in a non-specific manner [3].

## Conclusion

The use of plant extracts are a popular method for synthesisng meta and metal oxide NPs. ZnO NPs in particular are increasingly used in the biomedical field due their unique physicochemical characteristics. The present study reported on the procedure for green synthesis of ZnO NPs using Musa Paradisiaca and a Rooibos and Buchu extract. The characterisation by UV-vis spectroscopy revealed maximum absorption at 290 nm while that of the Scanning Electron Microscope revealed rod-like structures. The GZnO NPs demonstrated antimicrobial activity against Candida albicans. There is an increasing demand for biosynthesized nanoparticles for a broad range of applications.

## Declaration of Competing Interest

The authors declare that they have no known competing financial interests or personal relationships that could have appeared to influence the work reported in this paper.

## Data Availability

Data will be made available on request.
